# Utility of CT texture analysis to differentiate olfactory neuroblastoma from sinonasal squamous cell carcinoma

**DOI:** 10.1038/s41598-021-84048-5

**Published:** 2021-02-25

**Authors:** Masaki Ogawa, Satoshi Osaga, Norio Shiraki, Daisuke Kawakita, Nobuhiro Hanai, Tsuneo Tamaki, Satoshi Tsukahara, Takatsune Kawaguchi, Misugi Urano, Yuta Shibamoto

**Affiliations:** 1 Department of Radiology, Nagoya City University Graduate School of Medical Sciences, 1, Kawasumi, Mizuho-cho, Mizuho-ku, Nagoya, 467-8601 Japan; 2 Clinical Research Management Center, Nagoya City University Hospital, Nagoya, Japan; 3 Department of Radiology, Nagoya City West Medical Center, Nagoya, Japan; 4 Department of Otorhinolaryngology, Head and Neck Surgery, Nagoya City University Graduate School of Medical Sciences, Nagoya, Japan; 5 Department of Head and Neck Surgery, Aichi Cancer Center Hospital, Nagoya, Japan; 6 Department of Radiology, East Nagoya Imaging Diagnosis Center, Nagoya, Japan; 7 Department of Radiology, Kariya Toyota General Hospital, Kariya, Japan

**Keywords:** Medical research, Neurology

## Abstract

The purpose of this study was to examine differences in texture features between olfactory neuroblastoma (ONB) and sinonasal squamous cell carcinoma (SCC) on contrast-enhanced CT (CECT) images, and to evaluate the predictive accuracy of texture analysis compared to radiologists’ interpretations. Forty-three patients with pathologically-diagnosed primary nasal and paranasal tumor (17 ONB and 26 SCC) were included. We extracted 42 texture features from tumor regions on CECT images obtained before treatment. In univariate analysis, each texture features were compared, with adjustment for multiple comparisons. In multivariate analysis, the elastic net was used to select useful texture features and to construct a texture-based prediction model with leave-one-out cross-validation. The prediction accuracy was compared with two radiologists’ visual interpretations. In univariate analysis, significant differences were observed for 28 of 42 texture features between ONB and SCC, with areas under the receiver operating characteristic curve between 0.68 and 0.91 (median: 0.80). In multivariate analysis, the elastic net model selected 18 texture features that contributed to differentiation. It tended to show slightly higher predictive accuracy than radiologists’ interpretations (86% and 74%, respectively; *P* = 0.096). In conclusion, several texture features contributed to differentiation of ONB from SCC, and the texture-based prediction model was considered useful.

Olfactory neuroblastoma (ONB) is a rare malignant neuroectodermal tumor, accounting for 2 to 6% of all nasal/sinonasal tumors^1,2^, and is most commonly characterized by intracranial extension through the cribriform plate or skull base with rapid growth^1,3^. The symptoms are nonspecific and similar to those of benign sinonasal masses such as polyps, which can lead to delays in diagnosis. The optimal treatment for ONB is complete tumor resection; a craniofacial surgical approach is often performed but is technically quite difficult^1,2^. The most important differential diagnosis for ONB is squamous cell carcinoma (SCC), which is the most common malignant sinonasal tumor and has similar clinical manifestations to ONB^2^. Endoscopic excisional biopsy is the gold standard for definitive diagnosis of sinonasal tumors, but its diagnostic sensitivity is not high because of surrounding inflammatory tissues, patterns of local spread, and massive nasal hemorrhage^2^. Noninvasive imaging techniques, such as computed tomography (CT) and magnetic resonance (MR) imaging, are performed first for a suspected sinonasal tumor, and malignant tumors are suspected based on a finding of bony destruction^2,3^. However, the differential diagnosis between ONB and SCC remains difficult.

Most ONBs are centered in the superior nasal cavity, ethmoid, or cribriform plate, and characteristic CT image features were reported to be a well-circumscribed margin, uniform enhancement, and bony erosion^1,3,4^. As a specific MR feature, a relatively rare finding of cysts along the intracranial margin of a tumor was reported^3,5^. Nevertheless, differentiation is difficult on conventional CT and MR images^2^. Xiao et al.^2^ reported the utility of diffusion kurtosis imaging and dynamic contrast-enhanced MR imaging for differentiation between ONB and SCC. However, these advanced techniques are not routinely performed in many hospitals. CT examinations are probably performed more often than MR owing to the shorter scan time, high spatial resolution with multiplanar reconstruction images, stable image quality with few artifacts, low cost, greater number of devices in the world, and lack of MR contraindications such as pacemakers. Several studies have recently reported that texture analysis of CT images, a post-processing method, can add finer structural information related to tumor grade and phenotype that is not visually evident on conventional morphological analysis^6–12^. Image texture is defined as a complex pattern within an image consisting of simpler sub-patterns with characteristic features that may be evaluated by quantitative analysis. Texture analysis allows mathematical evaluation of tumor heterogeneity that is related to differences in pathologic features not easily quantifiable by the human eye^6–12^. The purpose of this study was to examine differences in texture features between ONB and SCC on contrast-enhanced CT (CECT) images and to evaluate the diagnostic performance of texture analysis.

## Methods

### Study design and patients

This retrospective study was approved by *Nagoya City University Graduate School of Medical Sciences and Nagoya City University Hospital Institutional Review Board* (No. 60-19-0114) and other four participating institutions, with a waiver for informed consent. All methods were performed in accordance with the relevant guidelines and regulations. ONB is a rare tumor, and we collected cases from several institutions in our university group. The privacy of the patients was carefully protected. The patients were selected following a search of medical records between May 2005 and February 2020. Eligibility criteria for entry were: (1) adult patients; (2) histological diagnosis of primary nasal and paranasal ONB or SCC; and (3) CECT examination reconstructed with 3-mm slice thickness before treatment. The exclusion criteria were: (1) recurrence, previously treated, or metastasis; and (2) CECT images affected by motion or streak artifacts. Consequently, 17 cases with ONB and 26 cases with SCC were fully analyzable.

### CT scanning

CT images were obtained using multidetector-row helical CT scanners. The number of detectors was either 16, 64, 128, or 320. CT images were acquired with the following protocol: spiral mode; slice thickness and reconstruction interval, 1 to 3 mm; matrix, 512 × 512; matrix size, 0.33 × 0.33 mm to 0.64 × 0.64 mm (field of view, 170 × 170 mm to 330 × 330 mm); and kernel, suitable for interpreting soft tissues. All axial images were reconstructed with 3-mm slice thickness. 1.1 to 2.0 ml/kg of body weight (maximum 100 ml) of a 300 mg I/mL nonionic iodinated contrast material was injected into a peripheral vein at a rate of 1.1 to 2.0 ml/s, and images were obtained at delays of 70 to 120 s.

### Image data analysis

**Figure 1:**
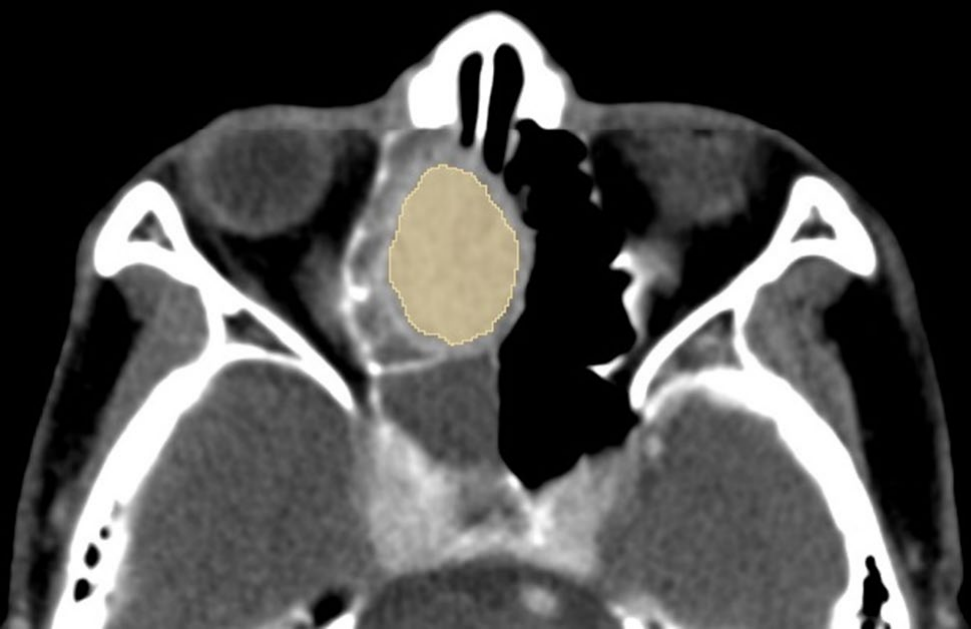
Representative example of a patient with ONB. The manually drawn ROI (in yellow) is delineated on an axial CECT image.

We used 3D Slicer (Version 4.1; www.slicer.org), an open source software package. In each slice, a radiologist with 11 years of experience (M.O.) manually drew a region of interest (ROI) on the visible tumor on axial CECT images, referencing sagittal and coronal images and non-contrast CT images. Clinical and pathological data were blinded. The ROI was drawn as large as possible with enough distance from the tumor edge to avoid a partial volume effect and we excluded voxels containing obvious non-enhanced cystic and necrotic areas, artifacts related to beam hardening, air, and calcifications, in accordance with previous studies^7,13–15^. Simply removing the slices with streak artifacts did not affect the performance of texture analysis in previous studies^16,17^. Figure 1 is a representative case in which the ROI of the tumor was manually drawn. Texture features from ROI were extracted using an extension of 3D Slicer software called SlicerRadiomics (version: 2.12; https://github.com/Radiomics/SlicerRadiomics)^8^. This extension included with the “Pyradiomics” library can calculate a variety of texture features based on Imaging Biomarker Standardization Initiative (IBSI) definitions^18^. In this software, an image pre-processing function is included. Hounsfield Unit values of voxels in CT images are used for calculations. Images were upsized to give symmetrical voxels of 1.5 mm using linear interpolation, because matrix size resampling was reported to be an appropriate preprocessing step in order to normalize differences of image data sets depending on different CT scanners and reconstruction settings in previous studies^17,19,20^. A total of 42 texture features for each tumor were extracted, applying the following criteria: first-order statistics (18 features) and Gray-Level Co-Occurrence Matrix (GLCM, 24 features). The first-order statistics assess the distribution of CT numbers or voxel values, such as mean, median, standard deviation, maximum, minimum, entropy, kurtosis, and skewness of the histogram. GLCM is a second-order statistic that assesses spatial relationships between adjacent voxels, providing measures of intra-lesion heterogeneity^8,14^. GLCM evaluates how frequently a pair of intensity levels is identified in an orientation based on a specified angle and radius. The co-occurrence matrix was determined for a distance of 1 pixel over 13 angle directions in 3D data. The value of a feature was calculated for each angle, and the mean of these values was outputted. A fixed bin width was used for gray discretization level which is equally spaced from 0. Mathematical definitions of these features are described in https://pyradiomics.readthedocs.io/en/latest/features.html.

### Radiologists’ interpretations

The CT images were reviewed by two radiologists (M.U. and T.K., both with 15 years of clinical experience), who were blinded to the pathologic and clinical information. The two radiologists visually classified all cases as either ONB or SCC, based on tumor localization, degree of enhancement, and heterogeneity. Decisions were reached by consensus, in accordance with previous studies^8,13^.

### Statistical analysis

Statistical analyses were performed using R version 3.6.1 (R Foundation for Statistical Computing, Vienna, Austria) with glmnet and pROC packages. In univariate analysis, texture features for ONB and SCC were compared using Welch’s *t* test. To adjust for multiple comparisons, we performed a false discovery rate (FDR) correction using the Benjamini and Hochberg method and adjusted *P* values. *P* < 0.05 was considered indicative of a significant difference. The receiver operating characteristics (ROC) curve analysis was performed, calculating area under the curve (AUC) to assess the prediction capability.

**Figure 2:**
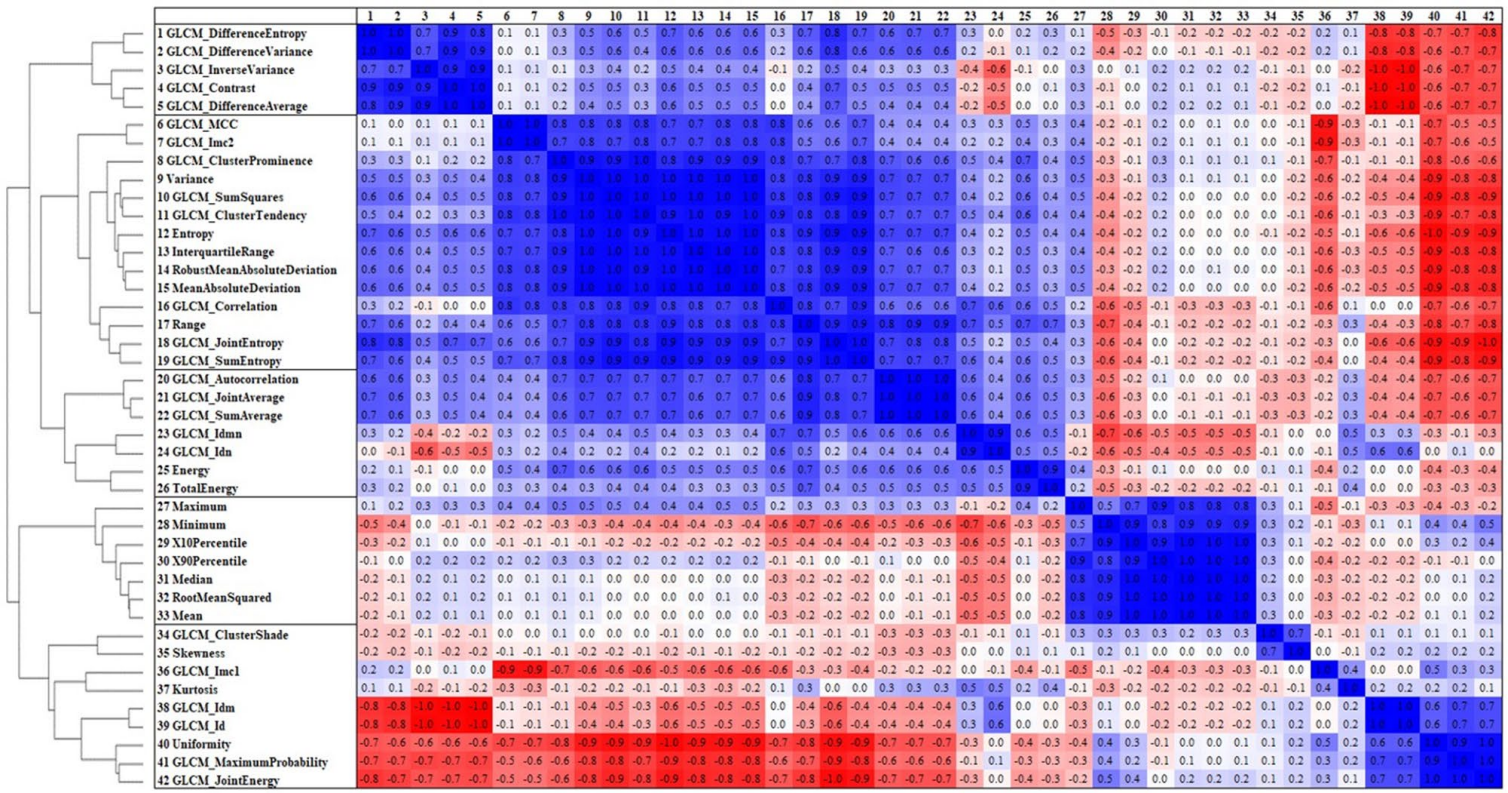
Cluster dendrogram (left) with heatmap (right) for 42 extracted texture features. Normalized Euclidean distance was used in hierarchical cluster analysis. More highly correlated clusters are located nearer to the bottom of the dendrogram. The features were sorted according to the result. Pearson correlation coefficients between respective features are shown in the heatmap; blue and red indicate negative and positive correlations, respectively. The heatmap and cluster dendrogram show a similar tendency.

In multivariate analysis, the elastic net^21^ was applied to select useful texture features and to construct a texture-based prediction model based on them. Normalization of variables was necessary for the elastic net and was automatically performed. The optimal hyperparameters of the elastic net, which are the mixing parameter of L_1_- and L_2_-penalties and regularization parameter, were determined to minimize leave-one-out cross-validation (LOOCV) error using grid search. Although the features and their coefficients of the final prediction model were determined using all subjects by the elastic net with the optimal hyperparameters, in order to avoid overestimating the prediction accuracy, LOOCV was also used to evaluate the performance^22^. As a similar regularization and automatic variable selection method, the least absolute shrinkage and selection operator (LASSO)^23^ was reported. However, if there is a group of variables among which the pairwise correlations are very high, the LASSO selects only one variable at random, and so it is inappropriate to identify multiple variables that contribute to differentiation^21^. Also, the maximum number of predictors in the LASSO is equal to the number of samples, and high correlations between predictors cause degraded prediction performance even if the number of predictors is smaller than the number of samples^21^. We hypothesized that some features might be highly correlated to each other due to the similarity of mathematical expression. We therefore performed correlation analysis for the extracted texture features (Fig. 2), and highly correlated features were seen. We chose the elastic net to overcome these limitations of LASSO^21^. Results of the correlation analysis were used as reference and we did not perform pre-selection of features. The accuracy between the texture-based prediction model and radiologist’s interpretations was compared using the McNemar test.

## Results

The patient and tumor characteristics of ONB and SCC are summarized in Table 1. There were no significant differences in the tumor diameter (*P* = 0.33). The radiologists interpreted that 15 of 17 cases with ONB and 8 of 26 cases with SCC were centered in the superior nasal cavity, ethmoid, or skull base.

**Table 1:** Patient and tumor characteristics of olfactory neuroblastoma and squamous cell carcinoma.

	Total (n = 43)	ONB (n = 17)	SCC (n = 26)	*P*-value
Age (mean ± SD), year	60 ± 14	54 ± 17	64 ± 11	0.10
Gender (male/female)	32/11	22/4	10/7	0.20
Longest diameter (mean ± SD), cm	4.5 ± 1.5	4.2 ± 1.3	4.6 ± 1.7	0.33
Centered in the superior nasal cavity, ethmoid, or skull base	23	15	8	0.18
Intracranial extension	9	7	2	0.01

*ONB* olfactory neuroblastoma; *SCC* squamous cell carcinoma, *SD* standard deviation.

In univariate analysis, significant differences were observed for 28 of the 42 texture features between ONB and SCC patients, with an AUC between 0.68 and 0.91 (median: 0.80). The *P* values, FDR-adjusted *P* values, AUC values, and cutoff point differentiating ONB from SCC for each feature are shown in Table 2.

**Table 2:** Texture features differentiating olfactory neuroblastoma from squamous cell carcinoma in univariate and multivariate analysis.

Texture feature	Univariate analysis				Multivariate analysis
	*P* value	FDR-adjusted *P* value	AUC Value	Cutoff point	Standardized regression coefficient
InterquartileRange	0.001	**0.003**	0.77	< 19	
Skewness	0.93	0.95	0.50		
Uniformity	0.005	**0.009**	0.76	> 0.45	
Median	0.000	**0.001**	0.84	> 93	**0.19**
Energy	0.030	**0.045**	0.78	< 2.3 × 10^6^	
RobustMeanAbsoluteDeviation	0.003	**0.005**	0.74	< 8.4	
MeanAbsoluteDeviation	0.001	**0.002**	0.78	< 12	**-0.071**
TotalEnergy	0.049	0.066	0.78		
Maximum	0.10	0.13	0.69		
RootMeanSquared	0.000	**0.001**	0.84	> 93	**0.17**
X90Percentile	0.001	**0.002**	0.82	> 111	**0.038**
Minimum	0.000	**0.001**	0.91	> 52	**0.36**
Entropy	0.001	**0.002**	0.79	< 1.4	**-0.13**
Range	0.000	**0.001**	0.85	< 69	**-0.12**
Variance	0.001	**0.002**	0.79	< 188	− **0.14**
X10Percentile	0.000	**0.001**	0.87	> 74	**0.32**
Kurtosis	0.18	0.23	0.61		
Mean	0.000	**0.001**	0.84	> 92	**0.19**
GLCM_JointAverage	0.002	**0.003**	0.79	< 2.5	
GLCM_SumAverage	0.002	**0.003**	0.79	< 5.0	
GLCM_JointEntropy	0.001	**0.002**	0.81	< 2.2	− **0.026**
GLCM_ClusterShade	0.69	0.79	0.56		− **0.26**
GLCM_MaximumProbability	0.036	0.052	0.70		
GLCM_Idmn	0.000	**0.001**	0.86	< 0.97	− **0.24**
GLCM_JointEnergy	0.007	**0.012**	0.77	> 0.21	
GLCM_contrast	0.63	0.73	0.55		
GLCM_DifferenceEntropy	0.028	**0.044**	0.70	< 0.99	
GLCM_InverseVariance	0.44	0.53	0.59		
GLCM_DifferenceVariance	0.047	0.065	0.70		
GLCM_Idn	0.001	**0.002**	0.79	< 0.92	− **0.17**
GLCM_Idm	0.89	0.95	0.52		
GLCM_Correlation	0.000	**0.001**	0.86	< 0.21	
GLCM_Autocorrelation	0.002	**0.004**	0.80	< 6.2	
GLCM_SumEntropy	0.000	**0.001**	0.84	< 2.0	− **0.10**
GLCM_MCC	0.028	**0.044**	0.68	< 0.39	
GLCM_SumSquares	0.001	**0.002**	0.79	< 0.41	− **0.005**
GLCM_ClusterProminence	0.002	**0.004**	0.83	< 2.0	− **0.005**
GLCM_Imc2	0.066	0.086	0.68		
GLCM_Imc1	0.92	0.95	0.53		
GLCM_DifferenceAverage	0.99	0.99	0.50		
GLCM_Id	0.81	0.90	0.52		
GLCM_ClusterTendency	0.000	**0.001**	0.82	< 0.89	− **0.042**
(Intercept)	–	–	–		− 0.45

*FDR* false discovery rate; *AUC* indicates area under the curve. Significant FDR-adjusted *P* values < 0.05 and non-zero standardized coefficients of the elastic net logistic regression are in bold.

**Figure 3:**
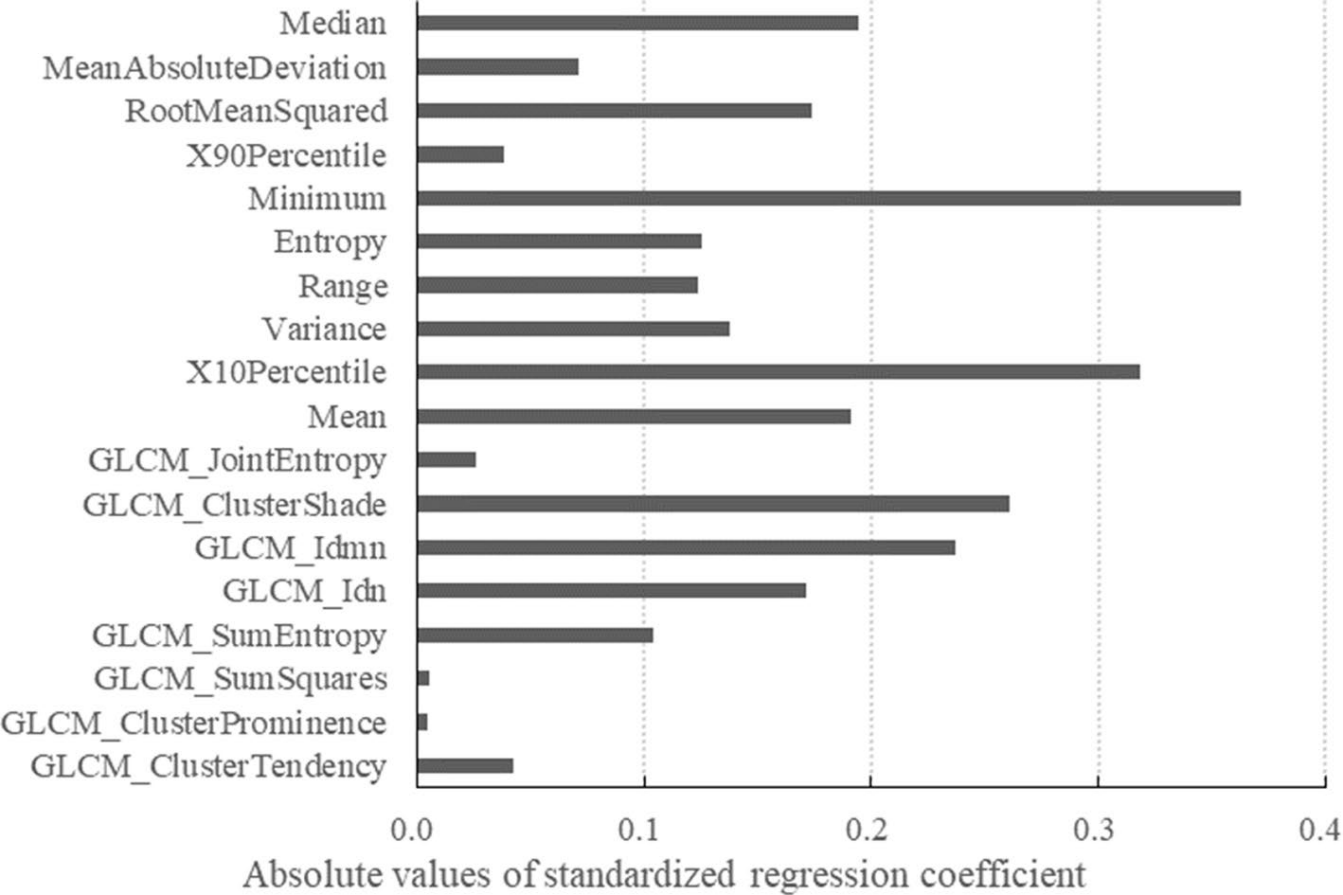
In multivariate analysis, texture features contributed to differentiation between ONB and SCC weighted by absolute values of standardized regression coefficients according to the elastic net logistic regression.

In multivariate analysis, the optimal mixing and regularization hyperparameters were respectively determined to be 0.44 and − 2.488 (logarithmic value) by the LOOCV method, and the number of selections for each feature in the 43 cross-validation models was tabulated in Supplementary Table 1. The feature-similarity between the cross-validation models evaluated by average Hamming distance was 2.08. The elastic net with the optimal hyperparameters identified a final predictive model with 18 texture features that contributed to differentiation, of which 17 showed significant differences in the univariate analysis. The linear predictor of the final model showed an AUC of 0.83. The standardized regression coefficients for these features and intercept are shown in Table 2. Figure 3 displays these features weighted by absolute values of standardized regression coefficient. Regarding prediction accuracy, the elastic net model and radiologists’ interpretations correctly classified 37 (86%) and 32 (74%) of the 43 cases, respectively. The elastic net model showed slightly higher predictive accuracy than radiologists’ interpretations, but there was no significant difference (*P* = 0.096). For SCC, the elastic net model also showed slightly higher accuracy than radiologists’ interpretations (96% and 77%, respectively), although the difference was not significant (*P* = 0.074). For ONB, both the elastic net model and radiologists’ interpretations showed accuracy rates of 71%. Detailed accuracy of the elastic net model and radiologists’ interpretations is shown in Table 3.

**Table 3:** Detailed accuracy of the elastic net model and radiologists’ interpretations.

	Accuracy rate				Elastic net	
	Radiologists (%)	Elastic net (%)			Correct	Wrong
Total (n = 43)	74	86	Radiologists	Correct	30	2
				Wrong	7	4
Olfactory neuroblastoma (n = 17)	71	71	Radiologists	Correct	10	2
				Wrong	2	3
Squamous cell carcinoma (n = 26)	77	96	Radiologists	Correct	20	0
				Wrong	5	1

## Discussion

Our study demonstrated the utility of texture analysis on CECT images in differentiating ONB and sinonasal SCC. In univariate analysis, significant differences were observed in 28 texture features. In multivariate analysis, the elastic net model selected 18 texture features that contributed to differentiation, and the prediction accuracy was 86%. LOOCV was used to avoid overestimating the prediction accuracy in multivariate analysis. One of the 18 features selected in multivariate analysis did not show significant differences in univariate analysis, due to differences in the analysis methods. The texture features were composed of first-order statistics assessing the distribution of CT numbers or voxel values and second-order statistics assessing spatial relationships between adjacent voxels^8,14^. Among several reported second-order statistic methods, we selected GLCM as it was the most frequently used in previous studies^6,7,9,13–15,19,22,24,25^. One previous study reported that the detectability of image heterogeneity might be superior with GLCM compared to first-order statistics^25^. Multiple features correlated to heterogeneity were included in the selected features, so uniform enhancement of ONB might be reflected. Nevertheless, it was difficult to discern how each mathematical texture feature was associated with the visual image pattern and underlying pathological tumor features. Especially in multivariate analysis, complicated relationships among each of the texture features make interpretation increasingly difficult, so detailed interpretation of the relationships between texture features and pathological features could not be made in many previous studies^6,8,9,13–15,20,22,24^. Our texture analysis did not yield information about tumor localization, in contrast to the radiologists’ interpretations. Most ONBs were centered in the superior nasal cavity, ethmoid, or cribriform plate, as previously reported^1,3,4^. Nevertheless, the texture-based prediction model showed high predictive accuracy, no less accurate than the radiologists’ interpretations, probably due to its objective assessment of the image pattern. Texture analysis might therefore help radiologists more accurately differentiate ONB and SCC centered in the superior nasal cavity or ethmoid.

Other rare superior nasal or ethmoidal tumors include undifferentiated carcinomas, neuroendocrine carcinomas, and small-cell undifferentiated carcinoma^1,2^. However, differentiation among ONB and these rare tumors is often difficult on conventional CT and MR images. In addition, the utility of advanced imaging is unproven^2^. Further studies using a greater number and larger variety of nasal and ethmoidal tumors are warranted to evaluate the possibility of differentiation among these tumors. As another differential diagnosis of sinonasal tumor, malignant lymphoma of the ethmoidal sinus is very rare^26^. In two previous large studies (n = 78 and 220), no lymphoma was found among malignant tumors of the nasal cavity and paranasal sinus^26^. Also, diagnosis of malignant lymphoma may not be difficult in most cases based on the typical image findings of strong diffusion restriction on MR images.

As a visual MR image feature, Som et al.^5^ reported that the finding of cysts along the intracranial margin of a tumor highly suggested ONB, although it was only seen in 3 of the 54 ONB cases. Texture analysis using MR images for other tumors was reported in a few studies^24,25^, but establishing the clinical utility and general diagnostic prediction model of MR texture features has been difficult in practice. The contrast, image noise, and artifacts of MR images are intricately affected by numerous factors: scan parameters, reconstruction parameters, difference between acquired and reconstructed matrix, hardware including multichannel coil, and vendor/version-specific reconstruction algorithm. Also, for 2D sequences commonly used in scanning the head and neck, the slice thickness and slice-selected direction differ depending on tumor size, tumor shape, and scanning time. 3D sequences require a longer scan time and the image quality is more significantly affected by these factors. There is currently no way to standardize these image differences affecting MR texture analysis, so all images should be obtained using the same protocol and MR scanner, which could result in a small sample size. Radiomics studies generally require a large number of image samples, typically obtained using different scanners and protocols^19^. In contrast, methods for standardizing differences of images for CT texture analysis were reported in previous studies: matrix size resampling and visual removal of slices with artifacts^16,17,19,20^. Consequently, CT texture analysis can be retrospectively applied to various clinical images, and CT images are more appropriate for establishing the utility of texture analysis and a general diagnostic model than MR images.

Our study had a few limitations. First, the small sample size and large number of extracted texture features may lead to model overfitting, limiting the generalizability of the results^13^. Nevertheless, ONB is very rare, so we used LOOCV for validation and the elastic net model to address this problem. The elastic net, an automatic variable selection and continuous shrinkage method, is useful when a relatively large number of predictors is found compared to the number of samples^21^. Also, our texture analysis method can be performed in other institutions for validation studies because we analyzed clinical images using an open-source software package based on IBSI, not a self-developed program. Second, the ROI in the tumors was manually drawn and voxels containing obvious non-enhanced cystic and necrotic areas were excluded. The presence of cysts is highly suggestive of ONB^3,5^, so it may be desirable to include cysts in the ROI. However, it might be difficult to distinguish cysts and necrosis visually on CT images, so both cysts and necrosis were excluded in our texture study.

In conclusion, several texture features of CECT images contributed to differentiation between ONB and SCC. The texture-based prediction model using the elastic net tended to show better predictive accuracy than radiologist’ interpretations, although the model did not incorporate tumor localization into the analysis.

## Supplementary Information

Supplementary Information
