# Supplementary material for: Utility of CT texture analysis to differentiate olfactory neuroblastoma from sinonasal squamous cell carcinoma
Source: Sci Rep. 2021 Feb 25;11:4679. doi: 10.1038/s41598-021-84048-5 (PMC7907098; doi:10.1038/s41598-021-84048-5)
Supplement: Supplementary file 1 — Supplementary Information [file 41598_2021_84048_MOESM1_ESM.docx]

**SUPPLEMENTARY INFORMATION**

**Utility of CT Texture Analysis to Differentiate Olfactory Neuroblastoma from Sinonasal Squamous Cell Carcinoma**

Masaki Ogawa^1^, Satoshi Osaga^2^, Norio Shiraki^3^, Daisuke Kawakita^4^, Nobuhiro Hanai^5^, Tsuneo Tamaki^6^, Satoshi Tsukahara^7^, Takatsune Kawaguchi^1^, Misugi Urano^1^, and Yuta Shibamoto^1^

^1^Department of Radiology, Nagoya City University Graduate School of Medical Sciences, Nagoya, Japan

^2^Clinical Research Management Center, Nagoya City University Hospital, Nagoya, Japan

^3^Department of Radiology, Nagoya City West Medical Center, Nagoya, Japan

^4^Department of Otorhinolaryngology, Head and Neck Surgery, Nagoya City University Graduate School of Medical Sciences, Nagoya, Japan

^5^Department of Head and Neck Surgery, Aichi Cancer Center Hospital, Nagoya, Japan

^6^Department of Radiology, East Nagoya Imaging Diagnosis Center, Nagoya, Japan

^7^Department of Radiology, Kariya Toyota General Hospital, Kariya, Japan.

**Supplementary Table 1.** Numbers of selections for features in cross-validation models.

| Texture feature | # of selections |
| --- | --- |
| InterquartileRange | 2 |
| Skewness | 0 |
| Uniformity | 0 |
| Median* | 43 |
| Energy | 1 |
| RobustMeanAbsoluteDeviation | 0 |
| MeanAbsoluteDeviation* | 43 |
| TotalEnergy | 1 |
| Maximum | 0 |
| RootMeanSquared* | 43 |
| X90Percentile* | 41 |
| Minimum* | 43 |
| Entropy* | 43 |
| Range* | 43 |
| Variance* | 43 |
| X10Percentile* | 43 |
| Kurtosis | 1 |
| Mean* | 43 |
| GLCM_JointAverage | 0 |
| GLCM_SumAverage | 0 |
| GLCM_JointEntropy* | 38 |
| GLCM_ClusterShade* | 42 |
| GLCM_MaximumProbability | 0 |
| GLCM_Idmn* | 43 |
| GLCM_JointEnergy | 0 |
| GLCM_Contrast | 0 |
| GLCM_DifferenceEntropy | 1 |
| GLCM_InverseVariance | 0 |
| GLCM_DifferenceVariance | 1 |
| GLCM_Idn* | 43 |
| GLCM_Idm | 0 |
| GLCM_Correlation | 10 |
| GLCM_Autocorrelation | 0 |
| GLCM_SumEntropy* | 43 |
| GLCM_MCC | 2 |
| GLCM_SumSquares* | 27 |
| GLCM_ClusterProminence* | 30 |
| GLCM_Imc2 | 0 |
| GLCM_Imc1 | 0 |
| GLCM_DifferenceAverage | 0 |
| GLCM_Id | 0 |
| GLCM_ClusterTendency* | 42 |

* Features selected in the final model.
